# Duodenal Traditional Serrated Adenoma: A Case Report and Literature Review

**DOI:** 10.7759/cureus.72635

**Published:** 2024-10-29

**Authors:** Ygor R Fernandes, Mateus Funari, Matheus Veras, Eduardo Guimarães De Moura

**Affiliations:** 1 Gastrointestinal Endoscopy, Faculdade de Medicina, Universidade de Sao Paulo, Serviço de Endoscopia Gastrointestinal do Hospital das Clínicas, Hospital das Clínicas da Faculdade de Medicina da Universidade de São Paulo (HCFMUSP), São Paulo, BRA; 2 Endoscopy, Hospital das Clínicas de São Paulo, São Paulo, BRA; 3 Gastroenterology, Faculdade de Medicina, Universidade de Sao Paulo, Serviço de Endoscopia Gastrointestinal do Hospital das Clínicas, Hospital das Clínicas da Faculdade de Medicina da Universidade de São Paulo (HCFMUSP), São Paulo, BRA

**Keywords:** duodenum, endoscopic resection, gastrointestinal neoplasia, malignant transformation, traditional serrated adenoma

## Abstract

Traditional serrated adenomas (TSAs) of the duodenum are rare lesions with potential for malignant transformation. We present the case of a 44-year-old female with a history of bariatric surgery who presented with worsening abdominal pain, nausea, and significant weight loss. Imaging studies, including MRI, revealed a 3.2 cm periampullary polyp in the second portion of the duodenum, with associated biliary dilatation. Endoscopic evaluation confirmed a pedunculated lesion with a villous surface, and en bloc resection was performed using a diathermic snare, followed by prophylactic hemostasis. Histopathology confirmed a TSA with low-grade dysplasia, with clear surgical margins. Postoperatively, the patient showed gradual recovery and remains asymptomatic on follow-up. Long-term endoscopic surveillance is planned due to the risk of recurrence or malignant transformation.

Duodenal TSAs, though infrequent, share molecular and histopathological characteristics with their colorectal counterparts. They are typically located in the periampullary region or the second portion of the duodenum. Complete endoscopic resection is often feasible, though larger lesions may require surgical intervention. Given the potential for recurrence or malignant progression, long-term surveillance is recommended. This case underscores the importance of early detection and appropriate management to prevent cancerous transformation.

Further studies are necessary to deepen the understanding of the natural history and optimal management strategies for duodenal TSAs, as current knowledge is largely extrapolated from colorectal TSA studies. This case contributes to the limited literature on duodenal TSAs and emphasizes the importance of vigilant follow-up.

## Introduction

Traditional serrated adenomas (TSAs) of the duodenum are exceedingly rare. The first documented case was in 2004, with only 36 additional cases reported since then [1]. TSAs are clinically significant due to their high potential for malignant transformation. In a review of duodenal TSAs, approximately 28.6% of cases were associated with invasive carcinoma, underscoring the aggressive nature of these lesions [2].

Duodenal TSAs are typically diagnosed in older adults, with a mean age of presentation around 70 years [2]. These lesions often present with nonspecific gastrointestinal symptoms such as abdominal pain, gastrointestinal bleeding, or anemia [3]. Endoscopically, they may appear as polypoid or sessile masses, typically located in the distal duodenum or around the ampulla of Vater [3]. Endoscopic management, including resection, has proven effective in the treatment of these lesions [4].

Histologically, TSAs are characterized by distinct serrated architecture and cytological atypia, including elongated, stratified nuclei and eosinophilic cytoplasm. Identifying these features is crucial for distinguishing TSAs from other serrated lesions, such as hyperplastic polyps or sessile serrated adenomas, which have different clinical implications [5]. Molecular analysis has revealed mutations in BRAF and KRAS, indicating their role in the serrated neoplasia pathway, which is similar to colorectal TSAs [6]. These findings are particularly relevant for African American populations, where molecular differences may alter clinical outcomes [7].

Recent studies have expanded the understanding of the molecular landscape of serrated lesions, identifying new mutations such as RNF43 and APC, alongside epigenetic alterations like methylation [8]. These molecular insights not only highlight the complexity of TSA pathogenesis but also suggest potential clinical applications for molecular testing, which could guide surveillance and therapeutic decisions [9].

Moreover, emerging literature suggests that duodenal TSAs may differ molecularly and clinically from their colorectal counterparts. While sharing key histopathological features, duodenal TSAs may follow distinct carcinogenic pathways, influencing their malignant potential and response to treatment [10]. Comparative studies between duodenal and colorectal TSAs remain limited, but they highlight the importance of understanding site-specific differences for optimizing management and improving patient outcomes [11].

Given the potential for malignant progression, the management of duodenal TSAs often involves complete endoscopic resection when feasible [4]. However, due to the lesion's location and size, surgical intervention may be required in some cases. The prognosis following resection is generally favorable, although long-term surveillance is recommended due to the risk of recurrence or the development of new lesions [2,4].

## Case presentation

A 44-year-old female with a history of bariatric surgery (Roux-en-Y gastric bypass) in 2012 and hypothyroidism presented with worsening abdominal pain, nausea, vomiting, and significant weight loss starting in late 2022. Initial imaging and an esophagogastroduodenoscopy in June 2023 revealed a stricture at the gastrojejunal anastomosis, which was suspected to be ischemic in origin. This led to a surgical revision of the anastomosis. Despite the surgical intervention, her symptoms persisted.

**Figure 1:**
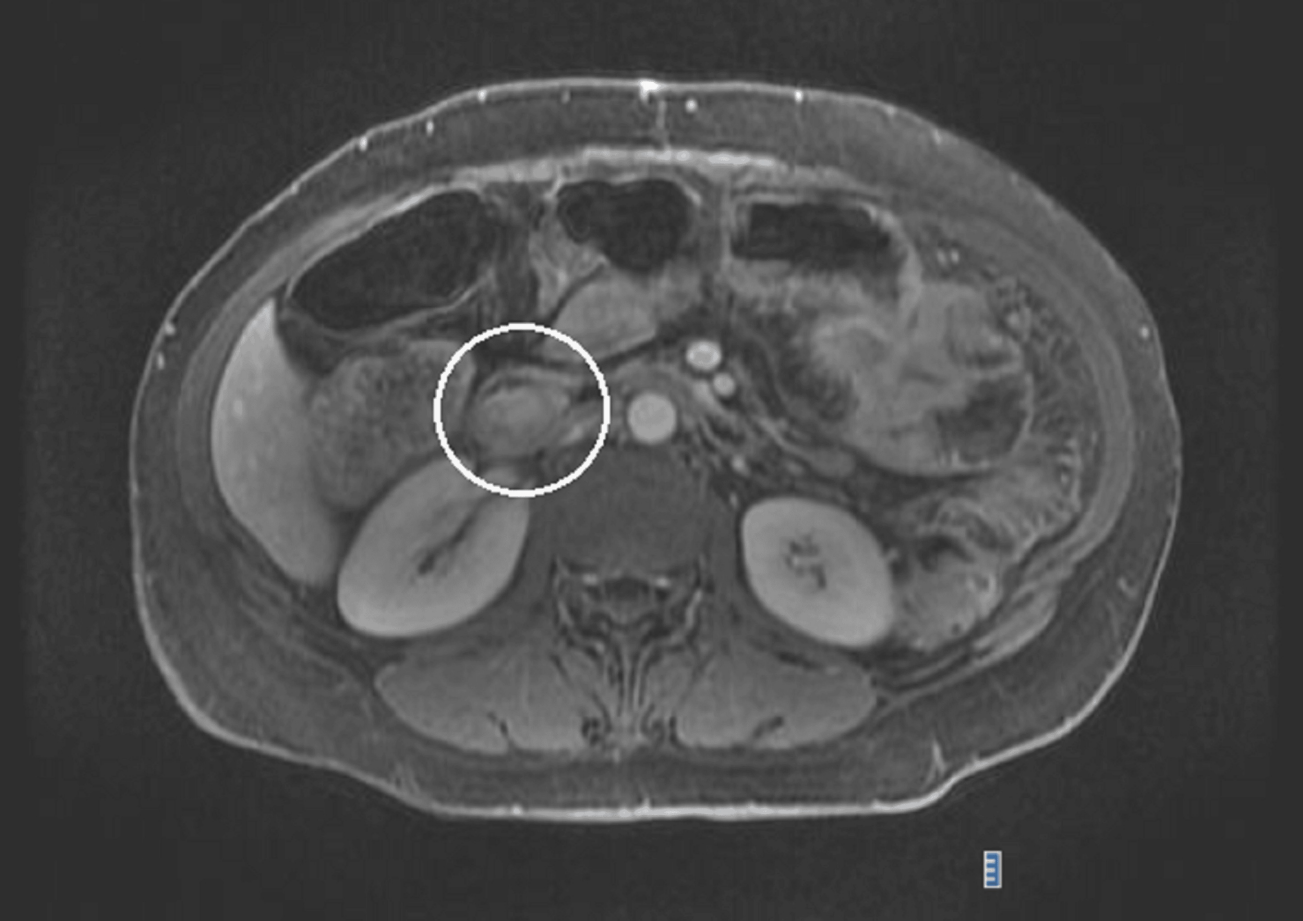
MRI of the upper abdomen MRI showing a well-defined, intraluminal polypoid lesion in the second portion of the duodenum adjacent to the papilla, measuring 3.2 cm in its greatest dimension. The lesion demonstrates heterogeneous enhancement, suggestive of vascularity, with no evidence of invasive features. Associated biliary dilatation is also observed, indicating possible obstruction. The distinct borders and absence of tissue invasion help differentiate this lesion from more aggressive malignancies. MRI: magnetic resonance imaging

In January 2024, a CT scan of the abdomen and pelvis showed an ulcerated gastrojejunal anastomosis with a blocked perforation and an associated collection, as well as a 2.5 cm periampullary polyp in the duodenum. To better characterize the lesion, an MRI of the upper abdomen was performed, revealing a 3.2 cm intraluminal polyp in the second portion of the duodenum with associated biliary dilatation (Figure 1). The MRI findings showed a well-defined, vascularized lesion with heterogeneous enhancement, which raised suspicion of a neoplastic process. However, the absence of invasive features on imaging suggested a benign etiology, leading to the decision to proceed with further diagnostic workup via endoscopy.

Laboratory findings prior to surgery included elevated inflammatory markers, suggesting an ongoing inflammatory or infectious process. Liver function tests revealed mildly elevated alkaline phosphatase and gamma-glutamyl transferase, consistent with biliary obstruction secondary to the periampullary polyp. The elevation in these markers highlighted the obstructive component of the patient's condition, necessitating prompt surgical and endoscopic intervention to prevent further biliary complications (Table 1).

**Table 1:** Summary of laboratory results and reference ranges

Parameter	Value	Reference range/normal values
C-reactive protein	35 mg/L	<5 mg/L
White blood cell count	12,000/mm³	4,000-11,000/mm³
Alkaline phosphatase	258 IU/L	44-147 IU/L
Gamma-glutamyl transferase	117 IU/L	9-48 IU/L

**Figure 2:**
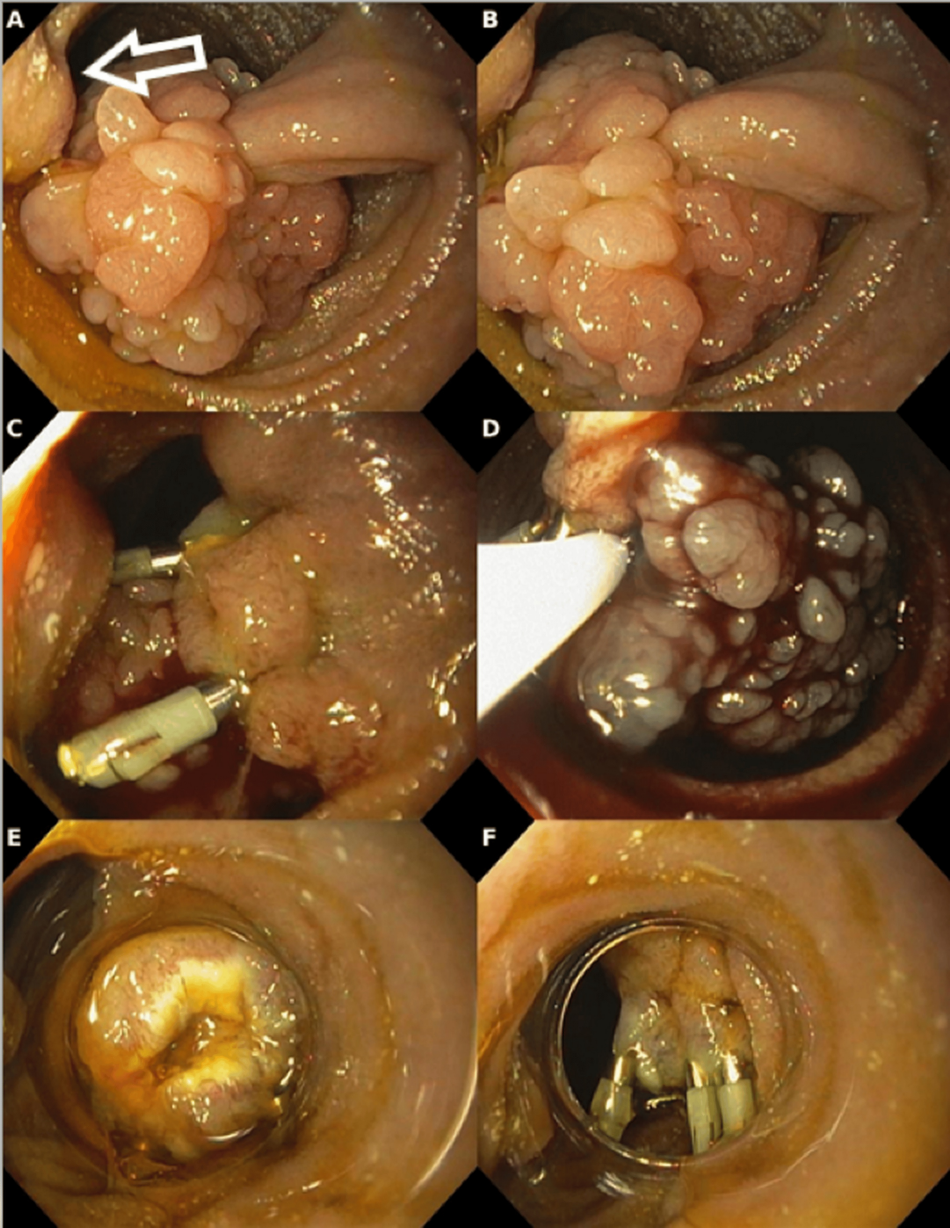
Endoscopic findings (A) Major duodenal papilla indicated by an arrow. (B) A pedunculated and exophytic lesion with a villous surface on the lateral wall of the second portion of the duodenum. (C) Two metal clips placed at the base of the lesion's stalk before resection. (D) En bloc resection using a diathermic snare. (E) Cap assistance to ensure optimized prophylactic hemostasis. (F) Final aspect after prophylactic hemostasis.

After a multidisciplinary discussion, the patient was scheduled for another surgery in March 2024 to address the anastomotic leak and peri-gastric collection. During this laparotomy, endoscopic access to the duodenum was achieved by gastrotomy. Endoscopic examination revealed a pedunculated, exophytic lesion with a villous surface, located on the lateral wall of the second portion of the duodenum, opposite the major duodenal papilla (Figure 2A-2B). The lesion measured approximately 30 mm in its cephalic portion, with a long (15 mm) and thick (12 mm) pedicle. The lobulated, villous appearance of the lesion suggested the possibility of a TSA, though malignancy could not be completely ruled out.

Given the lesion’s size and location, two metallic clips were placed at the base of the pedicle for prophylactic hemostasis (Figure 2C-2D). The lesion was then resected en bloc using a diathermic snare, with no residual lesion observed (Figure 2E). Two additional metallic clips were placed to ensure complete hemostasis (Figure 2F), as further endoscopic access to the duodenum would not be possible after the surgical closure.

Endoscopic resection was chosen over surgical resection due to several factors. The lesion, although measuring 3.2 cm, had a pedunculated and well-defined appearance, which made it amenable to en bloc removal using a diathermic snare. Also, endoscopic resection is often preferred when the lesion is accessible and does not present features suggestive of deep tissue invasion, as seen in this case with no evidence of malignancy on imaging or endoscopy.

The location of the lesion in the second portion of the duodenum, although challenging due to proximity to the ampulla of Vater, did not warrant surgical intervention given the absence of invasive characteristics. Moreover, the patient’s prior history of bariatric surgery complicated further invasive procedures, making endoscopic management a less morbid option. The decision to use metal clips for hemostasis was prompted by the lesion’s size and vascularized pedicle, increasing the risk of post-procedural bleeding. The placement of two metal clips at the base of the pedicle provided prophylactic hemostasis, preventing potential complications in an area that would become inaccessible after the surgical closure of the gastrotomy.

**Figure 3:**
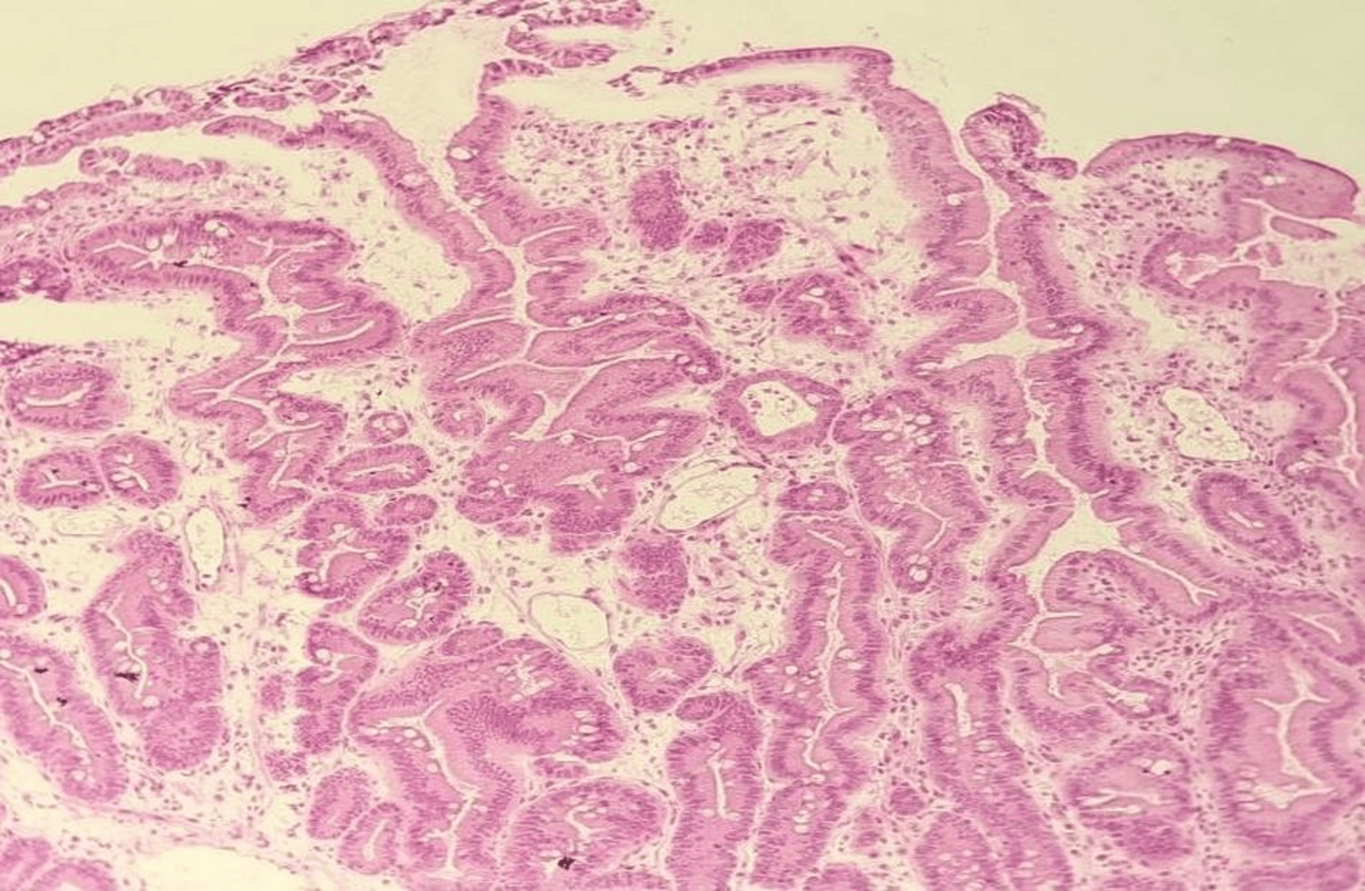
Histological examination of the resected TSA (H&E, 10x magnification) The histological section demonstrates the characteristic serrated architecture of a TSA, with elongated, stratified nuclei, and mild nuclear pseudostratification. Ectopic crypt formations and microtubular dysplastic structures are visible, differentiating the lesion from hyperplastic polyps or sessile serrated adenomas. TSA: traditional serrated adenoma, H&E: hematoxylin and eosin

Histopathological examination of the resected lesion confirmed the diagnosis of a TSA with low-grade dysplasia. The slide (Figure 3) reveals the characteristic serrated glandular architecture, with elongated and stratified nuclei, as well as mild nuclear pseudostratification.

## Discussion

TSAs of the duodenum are rare, with fewer than 40 cases reported in the literature [1]. This rarity limits our understanding of their long-term behavior and highlights the need for more focused studies [1]. In our case, a 44-year-old female presented with a 3.2 cm periampullary polyp located in the second portion of the duodenum, which was successfully resected endoscopically [3]. The lesion exhibited a pedunculated structure with a villous surface, consistent with previously reported cases of duodenal TSAs [1,2]. While some cases present with gastrointestinal bleeding [4], our patient's primary symptoms were abdominal pain, nausea, and weight loss [2]. These variations in clinical presentation underscore the importance of thorough endoscopic and histological evaluation to guide treatment decisions [5].

The malignant potential and recurrence rates of duodenal TSAs remain poorly defined due to the limited number of cases [2]. Studies have reported a low recurrence rate after complete resection, aligning with our patient’s outcome thus far [2,5]. However, the small sample sizes in these studies limit generalizability, emphasizing the need for more extensive longitudinal studies [6]. Given that approximately 28.6% of duodenal TSA cases have been associated with invasive carcinoma [1], regular follow-up remains essential, even when histology shows low-grade dysplasia, as in our patient [2]. The absence of recurrence in our patient so far is encouraging, but long-term surveillance will be crucial to ensure there is no progression [5].

Considering the malignant potential of these lesions, IHC plays a crucial role not only in confirming the diagnosis but also in providing prognostic information that may influence follow-up protocols [6]. In our case, the expression of MUC5AC and MUC6 helped confirm the diagnosis of TSA [5]. These markers, commonly associated with gastric-type mucin, are valuable in differentiating TSAs from hyperplastic polyps and sessile serrated lesions, which typically do not exhibit the same staining patterns [4]. Furthermore, the low Ki-67 index observed in our case supported the low-grade nature of the lesion [6], reducing the immediate concern for aggressive behavior [5]. In future cases, incorporating molecular testing, such as BRAF or KRAS mutation analysis, through IHC may provide a more comprehensive understanding of the lesion’s malignant potential [7]. While BRAF mutations are commonly identified in colorectal TSAs [6], their role in duodenal lesions remains less clear and warrants further investigation [7].

There are several limitations to this case report. The rarity of duodenal TSAs limits the broader applicability of our findings [3]. Another key limitation is the lack of multicentric studies with larger cohorts to validate management approaches and recurrence predictions for duodenal TSAs [6]. Furthermore, the absence of molecular testing in our case prevents a full analysis of the lesion’s genetic makeup, which could provide insights into its malignant potential [8]. Future case series should aim to incorporate both molecular and immunohistochemical profiles to better understand the relationship between these markers and clinical outcomes [8]. Additionally, larger multicenter studies are needed to establish standardized management protocols and surveillance guidelines for duodenal TSAs, as the available data remains scarce [9].

Given the lack of robust guidelines for post-resection surveillance of duodenal TSAs, our case highlights the necessity of individualized follow-up plans, particularly in light of the lesion's low-grade dysplasia and the patient's clinical stability [6]. Current recommendations are largely extrapolated from colorectal data [10], underscoring the need for specific studies on duodenal TSA recurrence and long-term outcomes [9]. In our case, the absence of recurrence so far is a positive indicator, but the patient will require long-term endoscopic monitoring to mitigate the risk of malignancy [6].

As molecular techniques advance, the integration of these methods in routine clinical practice will likely provide more precise risk stratification [7], enabling personalized management plans for patients with duodenal TSAs [8]. Future studies should focus on identifying molecular markers that can predict recurrence or malignancy, as well as defining the most appropriate surveillance intervals after resection [9]. Additionally, multicenter case series could provide more robust data on the prognosis and recurrence rates, aiding in the development of clear clinical guidelines [10]. A deeper understanding of the immunohistochemical and molecular profiles of duodenal TSAs will also be critical in guiding therapeutic decisions, particularly when endoscopic resection may not be feasible [11].

## Conclusions

This case contributes to the limited but growing body of literature on duodenal TSAs, emphasizing the role of endoscopic resection and immunohistochemistry in diagnosis and management. The successful removal of the lesion in this case, coupled with careful immunohistochemical evaluation, highlights the importance of personalized treatment strategies based on the lesion's size, location, and histopathological features. Future research, particularly on the molecular characteristics of these lesions, will be essential for optimizing the diagnosis and management of duodenal TSAs.
